# A non-linear beta-binomial regression model for mapping EORTC QLQ- C30 to the EQ-5D-3L in lung cancer patients: a comparison with existing approaches

**DOI:** 10.1186/s12955-014-0163-7

**Published:** 2014-11-12

**Authors:** Iftekhar Khan, Stephen Morris

**Affiliations:** Cancer Research UK & UCL Cancer Trials Centre, Cancer Institute, University College London, 90 Tottenham Court Road (5th floor), London, W1T 4TJ UK; Department of Applied Health Research, University College London, 1-19 Torrington Place, London, WC1E 7HB UK

**Keywords:** Mapping, Health economics, Lung cancer, Quality of life, Cross-walking, EORTC-QLQ-C30

## Abstract

**Background:**

The performance of the Beta Binomial (BB) model is compared with several existing models for mapping the EORTC QLQ-C30 (QLQ-C30) on to the EQ-5D-3L using data from lung cancer trials.

**Methods:**

Data from 2 separate non small cell lung cancer clinical trials (TOPICAL and SOCCAR) are used to develop and validate the BB model. Comparisons with Linear, TOBIT, Quantile, Quadratic and CLAD models are carried out. The mean prediction error, R^2^, proportion predicted outside the valid range, clinical interpretation of coefficients, model fit and estimation of Quality Adjusted Life Years (QALY) are reported and compared. Monte-Carlo simulation is also used.

**Results:**

The Beta-Binomial regression model performed ‘best’ among all models. For TOPICAL and SOCCAR trials, respectively, residual mean square error (RMSE) was 0.09 and 0.11; R^2^ was 0.75 and 0.71; observed vs. predicted means were 0.612 vs. 0.608 and 0.750 vs. 0.749. Mean difference in QALY’s (observed vs. predicted) were 0.051 vs. 0.053 and 0.164 vs. 0.162 for TOPICAL and SOCCAR respectively. Models tested on independent data show simulated 95% confidence from the BB model containing the observed mean more often (77% and 59% for TOPICAL and SOCCAR respectively) compared to the other models. All algorithms over-predict at poorer health states but the BB model was relatively better, particularly for the SOCCAR data.

**Conclusion:**

The BB model may offer superior predictive properties amongst mapping algorithms considered and may be more useful when predicting EQ-5D-3L at poorer health states. We recommend the algorithm derived from the TOPICAL data due to better predictive properties and less uncertainty.

**Electronic supplementary material:**

The online version of this article (doi:10.1186/s12955-014-0163-7) contains supplementary material, which is available to authorized users.

## Introduction

Mapping is a method where the interrelationship between a generic health related quality of life (HRQoL) measure such as the EuroQol EQ-5D-3L (EQ-5D-3L) and a condition specific HRQoL measure (e.g. EORTC QLQ-C30) is modelled so that utilities can be predicted (retrospectively) in studies where the generic measure was not used. Responses from the EORTC QLQ-C30 (QLQ-C30 thereafter) cannot be used directly in an economic evaluation because they are not measures of utility, although these can be obtained from external studies or algorithms. Therefore, a key objective of mapping is to estimate patient level utilities from which quality adjusted life years (QALY’s) are determined which might otherwise not be available. The EQ-5D-3L is recommended by the national institute for health care excellence (NICE) in the UK for use in economic evaluation, in particular, cost utility analyses (CUA) [1].

### Why use mapping

Mapping or “cross-walking” can be useful when patient level utilities are not available in a clinical trial. A statistical model, sometimes termed as ‘mapping algorithm’ is used to predict the EQ-5D-3L from a disease specific measure such as the QLQ-C30. If patient level EQ-5D-3L cannot be obtained then it becomes difficult to conduct a cost utility analysis with patient level data and reliance is made on published aggregate utilities. Mapping may therefore be the only way to estimate patient level utilities for a trial and can avoid the potential biases and uncertainties associated with using published aggregate utilities. Mapping can also offer an additional way of addressing sensitivity of estimated utilities (and QALYs) if there is concern about differences between the target population and the population from which utilities were estimated from valuation.

HRQoL outcomes from the QLQ-C30 are not based on *preferences* for given health states from a payer perspective, and therefore not used in CUA. However, a health state on the EQ-5D-3L of 12111 (for example, a score of 1 for Mobility, 2 for Self Care, 1 for Usual Activities, 1 for Pain/Discomfort and 1 for Anxiety/Depression) can be converted into a preference based utility value of 0.85 for generating a QALY.

The EQ-5D-3L currently used in many clinical trials, has 243 health states; for each state a corresponding utility value is available [2]. In this paper we use the UK tariffs based on the Time Trade-Off (TTO) method [3]. The raw scores from the EQ-5D-3L are converted into an index ranging from -0.59 to 1, where 1 denotes ‘perfect’ quality of life, 0 for death and values below 0 as states ‘worse than death’.

It is not uncommon to find a mapping algorithm used to predict patient level utilities for a clinical trial in a particular disease area (e.g. pain), although the mapping algorithm was developed using data from quite a different patient population [4]. Crott (2012) suggests that different algorithms or functional forms may exist for each cancer type [5]; similar issues have also been raised elsewhere [6]. Therefore, it is not often clear whether published mapping algorithms intend users to extrapolate to *any* patient population and how factors such as timing of measurements or presence of differential treatment effects influence predicted values. For example, where algorithms have been developed using only baseline data, it is often not immediately clear how useful they are for predicting post baseline EQ-5D-3L; users of algorithms are often interested in predicting differential (post baseline) treatment effects for cost-effectiveness purposes [7].

Although the EQ-5D-3L is a relatively short instrument, it is nevertheless surprising that many studies do not collect patient level utility data. Recently, only 16% of studies in lung cancer collected preference based HRQoL data [8]. In some clinical trials, patient level utilities were not collected, although formal economic evaluations were conducted; 25% of HTA submissions to NICE used mapping in such situations [9]. In Australian HTA’s, this was 24% [10]. There is no guarantee that future trials will continue to collect utilities from EQ-5D-3L as there is no obligation to do so. Moreover, the existence of the EQ-5D-5L (measured on a 5 point scale) suggests that the EQ-5D-3L may not be adequate to address concerns about sensitivity. These are some of the reasons why mapping may continue to play an important part in economic evaluation of new treatments.

In some early phase trials, preference based measures are not usually collected, but condition specific measures such as the QLQ-C30 are collected to provide early indications of symptom control for future phase II/III trial planning, particularly for re-imbursement. A mapping function can be used to estimate EQ-5D-3L from (combined) early trial data for planning future cost-effectiveness argument for a phase III trial. In situations where two identical trials are required for licensing purposes (e.g. multiple sclerosis), a useful mapping algorithm from one trial can be used to determine utilities in the other trial [11]. If the two trial designs and patient populations are identical, there might be a possibility for developing a mapping algorithm in the first trial and predict utilities for the second.

Although mapping can be useful (and sometimes necessary), it is preferable to collect EQ-5D-3L prospectively where possible [6]. Some particular problems identified with mapping include limited ability of models to predict utility at poorer health states (Rowen & Brazier [12]; the assumption that there is a conceptual relationship (overlap) between two measures (Round [13]) and that the predicted value is a true measure of HRQoL (Chuang [14]).

### Previous work on mapping

Several models have been developed and published for mapping the QLQ-C30 to predict EQ-5D-3L [15-19]. These models have been compared using measures such as predictive power (R^2^), predictive mean and residual mean squared error (RMSE). Models used for mapping include conditional mean or median regression models with varying degrees of success [6]. Of 119 models examined, ordinary least squares (OLS) approaches were the most common [6]. The authors concluded that more complex models only rarely had an impact on predicted values [6,20].

Response mapping approaches Gray et al [21] where ordinal categorical responses are modelled have also been used with limited success. With such models, however, if there are a few responses at extremes, prediction at these extremes are likely to be imprecise, particularly with smaller sample sizes. Hernandez et al [22] compare linear and TOBIT models with adjusted censored models (which essentially modify the TOBIT) and similar models in a mixture modelling framework (but none were applied to cancer datasets and QLQ-C30 in particular). These models appear to work well, but seem to need larger sample sizes to work well. For smaller clinical trials (such as the SOCCAR trial), these models too may have limited ability to predict at the extremes. Basu & Manca (2012) use a bayesian form of a beta regression (using non informative priors) in a non-mapping context and compared with OLS regression [23]. The authors used a 2-part version of the beta model to handle the over dispersion of values (e.g. over dispersion of 1′s on the EQ-5D-3L scale) [23]. Some relatively recent approaches include more complex Bayesian networks (Quang et al [24]) where conditional and joint probabilities of responses using Bayes theorem may compute posterior probabilities of each EQ-5D-5L response and consequently the expected utilities [24]. However, in this model too, predictions at poor health states were reported as inadequate, although it performed better than other models. The predicted utilities would be dependent on the initial (prior) probability of response for each EQ-5D-3L domain.

Longworth (2013) suggests several alternative models including the Beta Binomial for investigation [9]. Crott (2012) also suggest research of more complex mapping algorithms as well as a need for greater validation [5].

One aspect of this ‘complexity’ might include adding several interaction and additional demographic variables, which might improve prediction, but on the other hand can result in an over complicated algorithm (e.g. rather than 15 QLQ-C30 variables, one may have a model with 30 factors, including interactions to predict EQ-5D-3L). Another way of thinking about ‘complexity’ might be a more complicated mathematical function, but with fewer independent variables.

One feature of almost all published algorithms, including those used in lung cancer (using QLQ-C30) has been the over-prediction of utilities in patients with poorer health states. The commonly used models do not appear to have properly addressed over-dispersion at the extremes of the distribution. The authors of several mapping algorithms suggest further research is needed to address the uncertainty of algorithms in a robust way. Some authors suggest that statistical evaluation may not be sufficient [25] and clinical indicators are needed to support the algorithm. For these reasons, and given that at the current time no mapping algorithm exists for this particular type of lung cancer group (elderly and unfit for chemotherapy), this research is needed.

### Alternatives to mapping

Other approaches to predicting or estimating utilities from the QLQ-C30 include using valuation studies (such TTO) [12]. This raises a question on the usefulness of mapping the QLQ-C30 if utilities can be readily determined from published literature. However, as pointed out earlier, differences in disease (cancer) types can yield significant differences in predicted utilities. In our studies in non small cell lung cancer (NSCLC) patients, the average survival time was 3 months and patients were unfit for chemotherapy. In addition, patients from the trial were from the UK with relatively consistent practices for palliative care (often given to these patients).

Mapping is preferred over valuation when there is an absence of robust evidence from literature. Even when published utility measures are available, however, care should be taken that estimates of utility for an economic evaluation reflect those expected in the target population. In some cases valuation methods may focus on a reduced set of questions. For example, Rowen et al (2011) determine utilities associated with the QLQ-C30 in a cancer population with better prognosis and substantially longer median survival. The authors retain the question about a “long walk” for assessing physical function. In lung cancer patients, responses about “short walks” are likely to be just as if not more relevant [12]. The purpose of this research is not to compare utilities from valuation based approaches with mapping algorithms, but rather compare the more common mapping algorithms.

The value of the Beta Binomial (BB) model as a useful mapping algorithm is its flexibility and ability to model skewed and multimodal data measured on a zero to one interval [26]. The modelling context allows for clustering of data (to model correlations within and between subjects) and is shown to reported more precise and efficient parameter estimates [26]. In situations where responses are over inflated at extremes (ceiling effects), it is particularly useful because one can attempt to model extreme values rather than omitting them or considering them as outliers. Moreover, effect sizes in terms of odds ratios may be more meaningful to decision makers (particularly clinicians) than absolute mean differences.

## Methods

Several mapping models applied to QLQ-C30 were identified. A useful recent review is provided by Longworth [9]. Of the five published algorithms which mapped QLQ-C30, four used linear models (OLS estimates) [20] and one used a Quadratic model [12]. Lung cancer data sets were used in two instances [18,19].

### Instruments

The EQ-5D-3L is a generic HRQoL instrument which is well documented [2]. It has 5 domains Anxiety/Depression, Mobility, Self-Care, Usual Activities and Pain/Discomfort measured on a 3 point scale from 1 to 3. The UK TTO tariff was applied to the raw EQ-5D-3L scores to convert into utilities [2]. The EORTC QLQ-C30 is an established instrument for measuring HRQoL in various cancers [27]. The QLQ-C30 has 15 domains, scored on a 0 to 100 scale. The scoring consists of 5 function scales: Physical Function (PF), Role Function (RF), Emotional Function (EF), Cognitive Function (CF) and social functioning (SF). There are also 9 symptom scales, Fatigue (FA), Nausea & Vomiting (NV), Pain (PA), Dyspnoea (DY), Insomnia (IN), Appetite Loss (AL), Constipation (CO), Diarrhoea (DI) and Financial Problems (FI); there is also a global health status score (QL). For the global health and function domains, high scores indicate better QoL. For the symptom domains, low scores indicate better symptoms.

### Data

Data were from two national (UK) NSCLC clinical trials. Each trial received local ethics approvals and research was conducted in compliance with the Helsinki declaration (details in references provided). The first trial (TOPICAL) was a randomized phase III trial in 670 lung cancer patients which compare erlotinib (n= 350) with placebo (n= 320) [28]. Both the EQ-5D-3L and QLQ-C30 were collected monthly from randomization (baseline) until death. The analysis was based on using data over the first 12 months because nearly all patients had died by then.

The SOCCAR trial was a phase II randomized NSCLC trial comparing sequential chemotherapy followed by radical radiotherapy (experimental arm) versus concurrent chemo-radiotherapy followed by chemotherapy in 130 patients (70 in Concurrent vs. 60 in Sequential) with inoperable stage III NSCLC and good performance status. EQ-5D-3L and QLQ-C30 were collected monthly from baseline for a period of at least 18 months in the SOCCAR trial [29].

### Developing and testing alternative models

Separate mapping algorithms were developed using data from each of the TOPICAL and SOCCAR trials using BB regression and five other models (Linear, TOBIT, Quantile, Censored Least Absolute Deviation (CLAD), and Quadratic regression) for comparison. The five models selected are among the common mapping models reported in a review of the literature on mapping (Brazier et al [6]). Estimated utilities from each model were compared using several criteria including: RMSE, predicted distributions, MAE, confidence intervals, R^2^, residual plots, proportion of predicted EQ-5D-3L outside the range -0.59 to 1.0, estimated QALYs and Monte-Carlo simulation. The performance of each model was compared using independent data from the SOCCAR trial. In addition, each model was fitted using data from SOCCAR then tested with data from the TOPICAL trial.

### Model specification and analysis methods

For each model, data were combined across time points and treatment groups following methods of previously reported mapping algorithms [15,19]. One reason advocated for pooling across all time points is because more health states can be modelled. The models compared were:

(I) Linear Mixed Effect Model
(II) TOBIT Mixed Effect Model
(III) Quadratic Mixed Effects Model following Crott [15]
(IV) Quantile Fixed Effects Model
(V) Censored Least Absolute Deviation (CLAD): Fixed Effects Model
(VI) Mixed Effects Beta Binomial Regression Model

The linear mixed model is a regression model with subject as a random term. The linear mixed and Quadratic models, model the mean of the EQ-5D-3L utility distribution. The quadratic model (III) of Crott (2010) was included because it is considered as a non-linear model with squared terms for some QLQ-C30 domain scores. The Quadratic model has PF, EF, SF SL and DI as squared terms, following Crott [15]. The TOBIT models the mean ‘plus’ the remainder of the distribution in a mixed effects context, however the slope parameter is adjusted by the probability of censoring. The censoring in the TOBIT can be from either below (censoring EQ-5D-3L to 0) or above (censoring EQ-5D-3L to 1). The BB models the distributions of EQ-5D-3L responses. Models (I) - (III) are not described in detail because a review of the common features of these and other models have been discussed elsewhere [6].

### (IV) quantile regression

In linear regression, estimation problems can exist when a response variable such as the EQ-5D-3L is skewed, truncated or discrete [20]. The regression line (in the case of one independent variable) passes through the *mean* values of the response (EQ-5D utilities), for given values of the independent variable. It assumes that the relationship between EQ-5D and QLQ-C30 will be the same (changes by a constant slope), even if patients have very low or very high QLQC-30 scores. QLQC-30 may be predictive of EQ-5D, but the predictions may be different for poor quality of life (low EQ-5D scores) compared to better quality of life (high EQ-5D). Quantile regression might therefore be useful for predicting utilities in specific groups of patients (e.g. with worse health states); that is it examines how the relationship between EQ-5D and QLQC-30 changes, depending on the values of EQ-5D, in this application.

In quantile regression, a line would pass through the *median* or a specific *quantile* of the response for specified values of the independent variable (QLQC-30 scores). Predictions from the Quantile regression model for individuals subject are estimates of the (conditional) median when τ= 0.5 in the regression equation below:

$$ {\mathrm{Y}}_{\mathrm{i}} = {\mathbf{X}}^{\tau}\ast {\boldsymbol{\upbeta}}_{\tau }+{\boldsymbol{\upvarepsilon}}_{\mathrm{i}} $$

Y_i_ are observed EQ-5D utilities for each subject, **X** is a matrix of 15 QLQC-30 values including an intercept column, **β**_**τ**_ is a vector of parameters associated with the 15 scores of the QLQ-C30 and ε_i_ is the absolute deviation which is to be minimized, known as the least absolute deviation (LAD) [30,31] for estimating values of **β**_**τ**_.The expected median EQ-5D, when τ= 0.5 is assumed to increase for increasing QLQC-30 scores. No distributional assumptions are made about ε_i_, whereas in linear regression, ε_i_ are assumed normally distributed with constant variance. In practice, one might get several regression lines, one for each quantile. The relationship might be stronger at any one of these quantiles.

In order to predict a patient level median EQ-5D-3L utility (Y_i_^*^) from 3 given scores (such as PF, RF and EF), for example, a quantile (linear) regression model when τ= 0.5 would be:

$$ {{\mathrm{Y}}_{\mathrm{i}}}^{*}={\mathrm{b}}_0+{\mathrm{b}}_1*\mathrm{P}\mathrm{F}+{\mathrm{b}}_2*\mathrm{R}\mathrm{F}+{\mathrm{b}}_3*\mathrm{E}\mathrm{F} $$

When the values of PF, RF and EF are all zero, Y_i_^*^ is an estimate of the sample median.

### (V) Censored Least Absolute Deviation (CLAD)

CLAD extends quantile regression with an emphasis on the 50^th^ percentile (when τ= 0.5). In addition, predicted estimates of EQ-5D utilities which are either >1 or between 0 and -0.59 are restricted (censored) to 1 and 0 respectively (Powell; Khan & Powell) [32,33]. The model is described below:

$$ \mathrm{E}\mathrm{Q}-5\mathrm{D}-3\mathrm{L}=\left\{\begin{array}{c}\hfill \begin{array}{cc}\hfill 0\hfill & \hfill -0.59<\mathbf{X}\boldsymbol{\upbeta } +{\boldsymbol{\upvarepsilon}}_{\mathbf{i}} < 0\hfill \end{array}\hfill \\ {}\hfill \begin{array}{cc}\hfill 1.0\hfill & \hfill \mathrm{if}\ \mathbf{X}\boldsymbol{\upbeta } +{\boldsymbol{\upvarepsilon}}_{\mathbf{i}} > 1.0\hfill \end{array}\hfill \\ {}\hfill \begin{array}{cc}\hfill \mathbf{X}\boldsymbol{\upbeta } +{\boldsymbol{\upvarepsilon}}_{\mathbf{i}},\hfill & \hfill \mathrm{elsewhere}\hfill \end{array}\hfill \end{array}\right. $$

The term **Xβ** refers to the predicted median EQ-5D-3L plus some deviations, ε_i,_ which are assumed to follow any distribution with a median of zero. The estimators of **β** are unbiased and consistent though not efficient [34]. Conditional medians are estimated at the patient level in a similar way as in (IV) except that estimates are restricted to lie between 0 and 1. The mean of all the individual (conditional) medians can be used as before for deriving QALYs. The mean is the statistic of choice for decisions relating to health technology assessment [35,36]. The population mean and median are approximately equal for normally distributed data.

### (VI) Beta binomial regression

The BB distribution is often used in probabilistic sensitivity analyses (PSA) in health economic modelling for utility measures such as the EQ-5D-3L [37]. One reason for use in PSA appears to be convenience of assuming a scale from 0 to 1 for utility (although there is no EQ-5D-3L tariff which is exactly equal to zero). In particular, the BB regression can model responses which are unimodal or bimodal with varying levels of skewness [38-40] ; utilities are often reported as having skewed or truncated distributions [20]; in addition, the BB estimates the mean of the distribution whereas some other models estimate the median. Therefore the BB approach may be a suitable model to test for developing a mapping algorithm.

An important feature of the BB approach is that mean predicted estimates of EQ-5D-3L can be estimated while restricting the range between 0 and 1. Although responses are required to be in the (0,1) interval, the BB can still be used in any interval (a, b) for a < b using the transformation Y-a/b-a. For example, if the observed EQ-5D-3L value is -0.1, then -0.1 – (-0.53)/1- (-0.53) would give a transformed value of 0.28. However, it may be difficult to correct for both asymmetry and heteroscedasticity resulting in difficult interpretations of parameter estimates in terms of the original response [41]. Moreover, there may also be potential difficulties in interpreting mean QALYs. For example, a utility of -0.34 transformed to a value of 0.124 generates a different interpretation of the QALY. One reasonable assumption in the use of the BB model is that observed values <0 are set equal to 0 because there were <0.5% of values with EQ-5D-3L responses <0 in each data set, hence, the potential for bias is likely to be small. A transformation was therefore not used.

Using the BB can be more complicated than linear models, depending on the need to model the variance. Without modelling the variance (over-dispersion), modelling mean response (EQ-5D-3L) as a function of the 15 QLQ-C30 variables requires using a simple logit function (because values are assumed to lie between 0 and 1), as in a logistic regression model.

#### Notation for beta binomial

A response variable (EQ-5D-3L) is assumed to follow a Beta (α,β) defined by:

1 $$ \mathrm{F}\left(\mathrm{y}\Big|\upalpha, \upbeta \right)=\left\{\varGamma \left(\upalpha +\upbeta \right)/\varGamma \left(\upalpha \right)\varGamma \left(\upbeta \right)\right\}*{\mathrm{y}}^{\upalpha -1}*{\left(1-\mathrm{y}\right)}^{\upbeta -1} $$

The mean and variance of a y of (1) are α/(α + β) and αβ/(α + β)^2^(α + β + 1), where α and β are the shape and scale parameters, respectively. The parameters α and β can be estimated from the observed mean and variance using the method of moments. For example, the values of β from the TOPICAL and SOCCAR data are <1 for EQ-5D responses, using the relationship:

$$ \upalpha =\upmu *\left[\left(\left(\upmu *\left(1-\upmu \right)\right)/{\upsigma}^2\right)-1\right] $$

$$ \upbeta =\left(1-\upmu \right)*\left[\right(\left(\upmu *\left(1-\upmu \right)\right)/{\upsigma}^2-1\Big] $$

When μ and σ^2^ are population mean and variance; sample estimates can be used as estimates for these.

The above description of the BB distribution in (1) is not useful for regression modelling and first requires re-parameterization (Ferrari & Cribari-Neto 2004), so that a response can be defined along with a set of predictors to form a regression model [42]. This is similar to simple linear regression where the responses are normally distributed with mean μ and variance σ^2^. In simple linear regression we express μ, the predicted mean in terms of a set of predictors x_1_….x_15_ (i.e. μ= a + bx_1_ + bx_2_ + …..b_15_x_15_ for the QLQ-C30).

If we set μ= α/α + β and ϕ = α + β in (1) then this becomes:

2 $$ \mathrm{f}\left(\mathrm{y}\Big|\upmu, \upphi \right)=\left\{\varGamma \left(\upphi \right)/\varGamma \left(\upmu \upphi \right)\varGamma \left(\left(1-\upmu \right)\upphi \right)\right\}*{\mathrm{y}}^{\upmu \upphi -1}*{\left(1-\mathrm{y}\right)}^{\left(1-\upmu \right)\upphi -1} $$

The expression in (2) is a beta distribution: y ~ Beta (μ, ϕ) and the mean, μ is expressed as a link function (to model the mean) in terms of some predictor variables. Typically, the link function g(μ)= **Xβ** is such that g(μ)= log(μ/1- μ). With this logit link function, the mean response is: μ= e^ω^/1+ e^ω^, where ω= α + **Xβ**. The value of μ is restricted to a 0 to 1 scale. In simple linear regression, g(μ)= μ.

The BB regression form for modelling EQ-5D-3L is:

$$ \mathrm{g}\left(\upmu \right)= \log \left({\upmu}_{\mathrm{i}}/1-{\upmu}_{\mathrm{i}}\right)=\upalpha +\mathbf{X}\boldsymbol{\upbeta }, $$

where the part log(μ_i_/1- μ_i_) are the transformed values of the EQ-5D responses to a logit scale. One main difference with the logistic regression model is that there is no need for responses to be dichotomous (the values μ_i_ are continuous).

In order to compute the patient level predicted EQ-5D-3L, the logistic function, with **X** as the set of independent QLQ-C30 variables with **β** as the parameter vector yields:

$$ {\upmu}_{\mathrm{i}}= \exp \left(\mathbf{X}\boldsymbol{\upbeta } \right)/\left\{1+ \exp \left(\mathbf{X}\boldsymbol{\upbeta } \right)\right\} $$

A second model (such as a log function) could also be used to model the dispersion in terms of a set of QLQ-C30 variables (not necessarily all 15 variables, because the variance might depend on some important ones).

The additional precision parameter ϕ, like the mean parameter, μ, involves an equation which relates the variance to a set of predictors. This equation often assumes a log function ln[h(ϕ)] (because the variance is >0). Therefore, h(ϕ) describes the relationship between the variance and W

$$ \mathrm{i}.\mathrm{e}.\mathrm{h}\left(\upphi \right)= \exp \left(\mathbf{W}\boldsymbol{\updelta } \right), $$

Since the relationship between variance may not depend on all 15 variables (in the case of QLQ-C30), these are labelled **W** with the corresponding parameters **δ**.

The addition of the second (precision) parameter, ϕ, allows greater flexibility to model any over dispersion of EQ-5D values [42].

Hence, two sets of equations are associated with the QLQ-C30 variables; one through the mean EQ-5D-3L and one through the variance. These (two) equations provide the basis to determine estimates of the parameters **β**.

The responses y (i.e. the EQ-5D-3L) are therefore Beta ([(g(μ), h(ϕ)], with likelihood function:

3 $$ \mathrm{L}\left(\boldsymbol{\upbeta}, \boldsymbol{\updelta}, \mathbf{Y},\mathbf{X},\mathbf{W}\right)=\varGamma \left( \exp \left(\mathbf{W}\boldsymbol{\updelta } \right)\right)/\varGamma \left(\mathrm{s}\right)\mathrm{G}\left(\mathrm{t}\right){\mathbf{Y}}^{\mathrm{s}-1}{\left(1-\mathbf{Y}\right)}^{\mathrm{t}-1} $$

where,

$$ \mathrm{s}= \exp \left(\mathbf{X}\boldsymbol{\upbeta } +\mathbf{W}\boldsymbol{\updelta } \right)/\left\{1+ \exp \left(\mathbf{X}\boldsymbol{\upbeta } \right)\right\} $$

$$ \mathrm{t}= \exp \left(\mathbf{W}\boldsymbol{\updelta } \right)/\left\{1+ \exp \left(\mathbf{X}\boldsymbol{\upbeta } \right)\right\} $$

If we compare the above function (3) with (1):

$$ \mathrm{F}\left(\mathrm{y}\Big|\upalpha, \upbeta \right)=\left\{\varGamma \left(\upalpha +\upbeta \right)/\varGamma \left(\upalpha \right)\varGamma \left(\upbeta \right)\right\}*{{\mathrm{y}}^{\upalpha}}^{-1}*{\left(1-\mathrm{y}\right)}^{\upbeta -1} $$

α-1= s-1 and β-1= t-1 in (3) relates the observed EQ-5D-3L (y values) with the QLQ-C30 predictors through both the mean and variance.

This form of parameterization allows a powerful way of modelling utilities not only for mapping, but in a generalized mixed modelling context for estimates of utilities and HRQoL which can be scaled to a 0,1 interval. This approach uses a non-linear mixed modelling approach where the general likelihood has been programmed directly using the SAS software (version 9.3) [40]. An example SAS code is provided in Appendix I for implementing the BB model.

One reasonable assumption we impose is that observed values <0 are set equal to 0. There were <0.5% of values with EQ-5D-3L responses <0 in each data set, hence, the potential for bias is likely to be small. A transformation was therefore discarded.

Although the BB regression can be very flexible, a limitation of the model is that observations existing at either 0 or 1 must be scaled away from these values. That is, when there are many 0 or 1 responses, estimation with a standard BB regression can become problematic. Therefore, a *zero-one inflated* BB model was used to account for this over-dispersion [40].

#### Testing algorithms with lung cancer data

For each of the models, the predicted and observed values were compared. Models were developed using the larger TOPICAL data set and validated with SOCCAR. The observed health states were ordered (from 11111 to 33333) and the mean predicted EQ-5D-3L was computed for each algorithm and plotted. In addition, we estimated the proportion of individual predicted EQ-5D-3L responses from each model within +5% to +30% of the observed EQ-5D-3L. Estimated utilities from each model were compared using model statistics described earlier.

#### Model checking and adequacy

In all models, adequacy of fit was considered using residuals, tests for homoscedasticity and Aikakes Information Criterion (AIC). The AIC was used to compare models for the *same* dataset.

#### Simulations

Monte-Carlo simulations (10,000) from a multivariate distribution for the EQ-5D-3L and QLQ-C30 scores using the method of Fleishman [43,44] with the observed correlation structure were carried out to assess uncertainty of predicted means from each model. The method of Fleishman uses higher order moments (Skewness and kurtosis) as a way of simulating data that approximates the sampling distribution. Each data set simulated contained 670 and 130 patients with 2038 and 1002 observations for TOPICAL and SOCCAR respectively.

#### Addressing over prediction of EQ-5D-3L at the ‘poorer’ health states

We investigated the over prediction at ‘poorer’ health states using a health state of 11321 (EQ-5D-3L utility 0.433) as a cut-off for ‘Poor’ and ‘Good’ health states in TOPICAL and 22222 (EQ-5D-3L utility 0.516) in SOCCAR. The selected health states cut-points were chosen because this is where the observed and predicted EQ-5D-3L values start to diverge.

#### Impact on QALY estimates

For each model, patient level QALYs were generated using model estimates of patient level utilities and multiplying them by observed survival times. For PSA, simulation was used to estimate the mean overall survival (OS), progression free survival (PFS) and post-progression survival. OS and PFS are important outcomes in cancer trials often calculated from the time from treatment allocation or randomization until death (OS) or disease progression (PFS). The exponential model was chosen to fit the empirical Kaplan-Meier curve for OS and PFS. Using the relationship: OS= 1*Log(1-x_i_))/λ,where x_i_ are randomly generated from a uniform distribution and λ is the observed hazard rate. In economic evaluation, the objective is to simulate survival times which approximate the mean OS and PFS (not the median).

For each realization the mean (area under the survival curve) OS and PFS were determined for each treatment group. For TOPICAL, there were no censored data and for SOCCAR, the censoring distribution was taken into account (because patients were still alive) such that simulations resulted in PFS < OS. The pre and post progression utilities were determined from each of the simulations described earlier. Hence a total of 10,000 mean OS, PFS, pre-progression and post-progression utilities were generated to determine QALYs. QALYs were estimated as weighted sums of pre-progression and post-progression mean EQ-5D-3L for each treatment group.

## Results

A total of 2038 and 1002 data points with 84 and 54 health states were observed for each of TOPICAL and SOCCAR respectively. The average (median) number of observations per health state were 3 for TOPICAL and 2 for SOCCAR. The most frequent health state in TOPICAL was 21222 (12%); For SOCCAR, the most frequent health state was 11111 (25%) followed by 21222 (8%). Patients in the SOCCAR trial had better performance status (Table 1) compared to TOPICAL patients. Less than 0.5% (3/2038 observations in TOPICAL and 1/1002 in SOCCAR) of EQ-5D-3L observations had values <0 (corresponding to 3 health states in TOPICAL and 2 health states in SOCCAR). The correlation between EQ-5D utilities and each of the 15 domains ranged from 0.32 (FI) to 0.69 (PF), suggestion that mapping was possible (some overlap in terms of responses was present).

**Table 1:** **Summary of health states and baseline characteristics**

	**TOPICAL (N= 670)**	**SOCCAR (N= 130)**
EQ-5D number of observations	2038	1002
Health states (range)	84 (11111, 33312)	54 (11111, 23223)
[EQ-5D Value] {number of HS <0}	[1, -0.043] {3 HS < 0}	[ 1, -0.028] {2 HS < 0}
Median number of observations per HS	3	2
Most Frequent HS	21222 (12%) [value = 0.62]	11111 (25%) [ value = 1]
ECOG	1-3	0-1
Median Age (years)	77	62
Disease Stage	IIIb-IV	IIIa-IIIb

HS: Health states.

**Figure 1:**
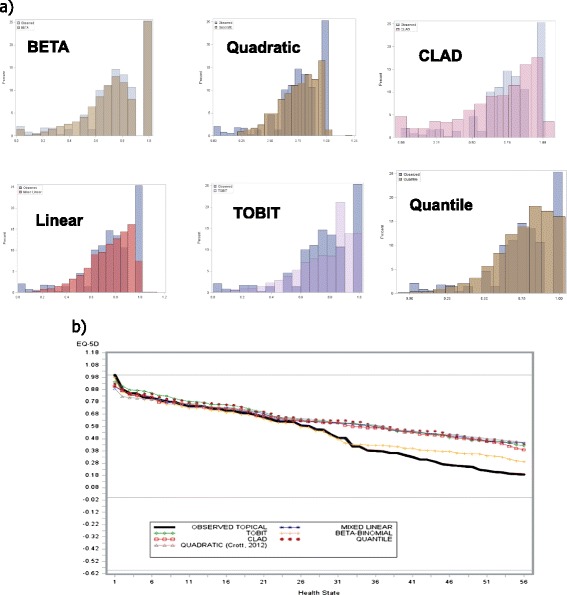
**Out of sample predictions. a)** Model developed from TOPICAL data tested on SOCCAR data. **b)** Model developed from TOPICAL data tested on SOCCAR data: Predictions by health states.

The observed mean (SD) EQ-5D-3L for TOPICAL and SOCCAR were 0.61 (0.29) and 0.75 (0.23) respectively over all post-baseline time points (Additional file 1: Table S1). Figure 1 shows the distributions of EQ-5D-3L confirming presence of non-normality, skewness and multimodality. If α and β (both are shape parameters which are used to model the distribution – for example if α and β are the same, the distribution tends towards symmetry) are <1, data are considered non-normal, multimodal or skewed. The Kolgomorov-Smirnoff goodness of fit rejects normality (P= 0.0093) and P < 0.001 for TOPICAL and SOCCAR respectively).

### Comparison of models

**Figure 2:**
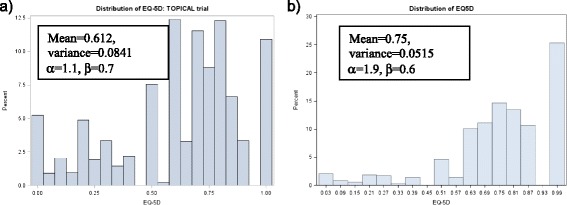
**Distribution of EQ-5D for TOPICAL and SOCCAR. a)** TOPICAL. **b)** SOCCAR.

The models were first tested on the same dataset used to develop the mapping algorithm (Table 2 and Additional file 1: Table S2, Figure 2a and 2b). All terms in the model were retained (regardless of statistical significance); even if some terms are not statistically significant, since they can still be relevant [5,12,16]. The standard errors for the BB were smallest (Table 2). The AIC values were smallest for the BB model and ranged from -2215 (BB) to -864 (Quantile) for TOPICAL and -1529 (BB) to -587 (Quantile). Smaller AIC values suggest better fit (Table 2). For the six models, the estimated R^2^ ranged from 0.53 (CLAD) to 0.75 (BB); R^2^ was highest (TOPICAL R^2^= 0.75) for the BB model (Table 2). Estimated RMSE ranged from 0.09 (BB) to 0.18 (Quadratic, CLAD). The proportion of predicted values of EQ-5D-3L >1 were highest for the quantile model (3.5%), whereas for the Quadratic model predicted values <0 were more common (5%), despite only 0.3% of observed values <0 were set to zero for modelling purposes (Table 2). Only the TOBIT, CLAD and BB did not predict outside the ‘observed’ range.

**Table 2:** **Summary of model fit statistics**

***QLQ-C30***	***Linear Mixed***		***TOBIT***		***Quadratic***		***Quantile***		***CLAD***		***Beta***	
	***TOPICAL***	***SOCCAR***	***TOPICAL***	***SOCCAR***	***TOPICAL***	***SOCCAR***	***TOPICAL***	***SOCCAR***	***TOPICAL***	***SOCCAR***	***TOPICAL***	***SOCCAR***
**R** ^**2**^	0.63	0.64	0.65	0.63	0.64	0.62	0.66	0.62	0.55	0.53	0.75	0.71
**MAE**	0.14	0.14	0.13	0.10	0.16	0.129	0.13	0.09	0.14	0.71	0.10	0.13
**RMSE**	0.183	0.141	0.17	0.14	0.18	0.14	0.17	0.14	0.18	0.15	0.09	0.11
Predicted mean (SE)*	0.584 (0.0047)	0.771 (0.0058)	0.631 (0.0057)	0.771 (0.0068)	0.635 (0.0074)	0.774 (0.0057)	0.633 (0.0054)	0.766 (0.0062)	0.593 (0.0059)	0.782 (0.0058)	0.608(0.0040)	0.749 (0.0049)
**Predicted >1 (%)**	0.11%	1.04%	0	0	0	1%	2.8%	3.5%	0	0	0	0
**Predicted <0 (%)**	0	0	0	0	5%	2%	0.6%	0.4%	0	0	0	0
**AIC (lower is better)**	−1015	−782	−936.9	−593	−978.6	−782	−864	−587	−926	−601	−2215	−1529
**SE of coefficients**												
**PF**	0.000264	0.000335	0.000288	0.000413	0.000686	0.000766	0.002805	0.000513	0.000295	0.000555	0.000190	0.000293
**RF**	0.000213	0.000231	0.000231	0.000285	0.000710	0.000635	0.000322	0.000267	0.000422	0.000287	0.000106	0.000220
**EF**	0.000214	0.000250	0.000233	0.000307	0.000532	0.000289	0.000312	0.000333	0.000392	0.000411	0.000289	0.000216
**SF**	0.000195	0.000203	0.000210	0.000249			0.000316	0.000263	0.000419	0.000313	0.000149	0.000204
**CF**	0.000207	0.000231	0.000225	0.000288			0.000244	0.000328	0.000447	0.000348	0.000163	0.000211
**FA**	0.000257	0.000296	0.000279	0.000373			0.000204	0.000373	0.000305	0.000391	0.000224	0.000251
**NV**	0.000234	0.000217	0.000255	0.000274			0.000255	0.000310	0.000255	0.000430	0.000258	0.000204
**PA**	0.000168	0.000203	0.000182	0.000250	0.000165	0.000200	0.000329	0.000410	0.000329	0.000460	0.000128	0.000211
**DY**	0.000154	0.000161	0.000168	0.000199			0.000201	0.000209	0.000201	0.000277	0.000159	0.000116
**SL**	0.000144	0.000165	0.000156	0.000203	0.000379	0.000277	0.000241	0.000211	0.000295	0.000291	0.000136	0.000149
**AP**	0.000138	0.000170	0.000150	0.000208			0.000242	0.000221	0.000292	0.000221	0.000129	0.000169
**CO**	0.000153	0.000158	0.000166	0.000196	0.000151	0.000155	0.000206	0.000207	0.000311	0.000283	0.000149	0.000206
**DI**	0.000152	0.000218	0.000165	0.000291	0.000405	0.000393	0.000219	0.000426	0.000213	0.000396	0.000133	0.000191
**QL**	0.000240	0.000294	0.000263	0.000372			0.000363	0.000210	0.000299	0.000260	0.000201	0.000241
**FI**	0.000204	0.000143	0.000224	0.000182			0.000101	0.000100	0.000209	0.000210	0.000243	0.000182

MAE: Mean Absolute Error; RMSE: Residual Mean Squared Error; SE: Standard Error.

*Observed post baseline Mean (SD) for TOPICAL was 0.61 (0.29) and for SOCCAR was 0.75 (0.23).

**Figure 3:**
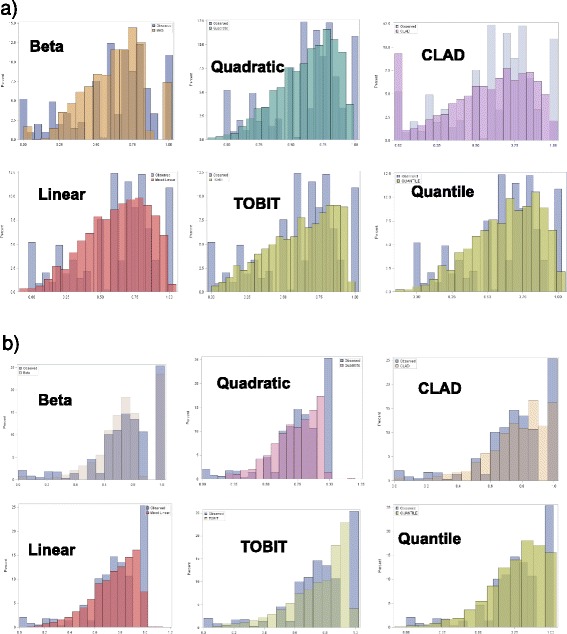
**Observed vs Predicted EQ-5D values. a)** TOPICAL Data.** b)** SOCCAR Data.

Mean predicted EQ-5D-3L distributions are also shown in Figure 3a and 3b. The predicted means were 0.608 for BB, 0.584 (Linear), 0.631 (TOBIT), 0.635 (Quadratic), 0.633 (Quantile) and 0.593 (CLAD) in TOPICAL; For SOCCAR these were 0.749 (BB), 0.771 (Linear), 0.771 (TOBIT), 0.774 (Quadratic), 0.766 (Quantile) and 0.782 (CLAD). Predicted mean EQ-5D-3L was closest to the observed with the BB model (Table 2).

### Testing models using independent data

The model developed from TOPICAL was tested on SOCCAR data (Figure 3a, 3b and Table 3). Figure 1 compares ‘out of sample’ predicted and observed EQ-5D-3L distributions. In particular, Figure 3a shows the predicted vs. observed distributions for the model developed from TOPICAL and tested using the SOCCAR dataset; the BB model predicts the over-dispersion at values of Zero and One better than other models: in SOCCAR, 25% of EQ-5D-3L responses were one: the BB has predicted these very well. The CLAD and Quantile also predict these with some success whereas TOBIT and Linear were less accurate. Figure 3b shows the predicted mean EQ-5D-3L by health state compared to observed values. The BB also over-predicts EQ-5D-3L at poorer health states, but the extent of the over-prediction is less severe.

**Table 3:** **Testing of models using independent data**

**Model**	**Predicted mean (SE) [95% CI]**	**R** ^**2**^	**RMSE**	**% of 95% CI containing the observed mean** ^**‡**^
Beta (a)*	**0.747 (0.0069)**	0.75	0.132	77%
	[0.733, 0.760]			
Beta (b)^†^	**0.622 (0.0057)**	0.61	0.159	59%
	[0.608, 0.631]			
CLAD (a)*	**0.671 (0.0091)**	0.56	0.027	28%
	[0.652, 0.689]			
CLAD (b)^†^	**0.652 (0.0054)**	0.47	0.154	19%
	[0.639, 0.660]			
Linear Mixed (a)*	**0.738 (0.0051)**	0.63	0.019	45%
	[0.728, 0.747]			
Linear Mixed (b)^†^	**0.642 (0.0059)**	0.58	0.095	23%
	[0.630, 0.653]			
Quadratic (a)*	**0.768 (0.0056)**	0.63	0.018	37%
	[0.757, 0.778]			
Quadratic (b)^†^	**0.636 (0.0039)**	0.55	0.093	14%
	[0.628, 0.643]			
TOBIT (a)*	**0.739 (0.014)**	0.56	0.021	65%
	[0.702, 0.757]			
TOBIT (b)^†^	**0.644 (0.0084)**	0.59	0.112	24%
	[0.627, 0.660]			
Quantile (a)*	**0.772 (0.0060)**	0.62	0.190	21%
	[0.754, 0.778]			
Quantile (b)^†^	**0.661 (0.0084)**	0.58	0.148	8%
	[0.644, 0.677]			

*Model developed from TOPICAL trial and tested using SOCCAR Data.

^†^Model developed from SOCCAR trial and tested using TOPICAL Data.

^‡^based on 10,000 monte-carlo simulations.

The R^2^ values were highest with the BB (R^2^= 0.75) when the model developed from TOPICAL data was tested on SOCCAR data and R^2^= 0.61 for the model developed from SOCCAR data and tested on TOPICAL; the RMSEs were higher compared with other models (Table 3). Mean predicted EQ-5D-3L for SOCCAR was 0.747 (95% CI: 0.733, 0.760) for the BB. Hence, the BB developed from TOPICAL data predicted mean EQ-5D-3L to within 0.4%. From 10,000 simulations, 77% of the 95% confidence intervals for the predicted mean EQ-5D-3L contained the observed SOCCAR mean EQ-5D-3L value of 0.75.

When the model was developed using SOCCAR data and then tested on TOPICAL, the mean predicted mean EQ-5D-3L was 0.622 (compared with the observed 0.61) and 59% of the 95% confidence intervals contained the mean EQ-5D-3L value of 0.61 observed in TOPICAL. On possible reason for the lower proportion of coverage is that SOCCAR patients had less severe NSCLC patients and consequently ‘better’ health states compared to patients in the TOPICAL trial. Normal probability plots of the model tested on SOCCAR data show residuals closest to the line with the BB model (Additional file 2: Figure S1).

#### Patient level predictions using independent data

Comparing mean predicted EQ-5D-3L with observed mean may not always be the best way to judge model performance because the distributions of the prediction values tend to cluster around the observed mean [6]. For example, an observed mean utility of 0.61 compares with a predicted mean of 0.593 using CLAD (Table 2), a difference of 0.017 (3%). However, about 40% of individual predicted values differed from the observed mean by about 10%. Therefore, for each patient, percent differences within ±5% to ±30% of observed values were calculated. About 28% (BB) of predicted EQ-5D-3L were within ±5% (Additional file 3: Figure S2) of the observed EQ-5D-3L compared to 20% (Linear), 23% (TOBIT), 24% (Quadratic), 22% (Quantile) and 22% (CLAD) with SOCCAR data. Predictions were in general better with the BB model (the curve is above all others). The median prediction error for the BB model is about 10% for both TOPICAL and SOCCAR. Highest prediction errors are observed with the Linear model (median of 15% error for both TOPICAL and SOCCAR). The QLQ-C30 responses ranged from 0 to 100 for 14 out of the 15 domains (scores for the financial domain ranged from 30 to 80).

### Over-prediction in worse health states

**Figure 4:**
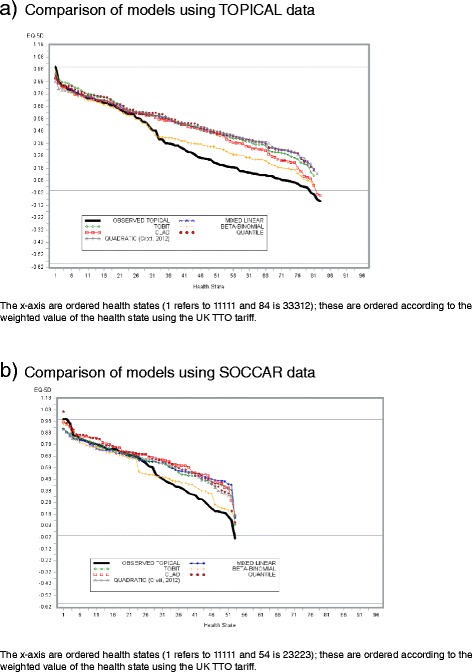
**Predicted EQ-5D versus observed EQ-5D for each Model by health state. a)** Comparison of models using TOPICAL data. **b)** Comparison of models using SOCCAR data.

Mean predicted EQ-5D-3L at observed health states for each algorithm are shown in Figures 4a and 4b. The BB model had mean predicted EQ-5D-3L estimates closest to the observed values at a given observed health state for TOPICAL and SOCCAR respectively. Differences between observed and predicted mean EQ-5D-3L for most models occur at about health states of11321 (value on x-axis of 32 in Figure 4a) for TOPICAL and about 22222 (x-axis value of 26) for SOCCAR. The Quadratic model under-predicted mean EQ-5D-3L at less severe health states compared to the BB model (Figure 4a). With SOCCAR data, the BB model approximates mean EQ-5D-3L at each health state better than all other models (Figure 4b). Figure 1b shows a similar plot using independent data from the TOPICAL developed algorithm.

#### Relationship between health states and adverse events

The relationship between adverse event frequency for different definitions of ‘Good’ and ‘Poor’ health states were briefly investigated. The results suggest that patients with ‘Poor’ health (defined roughly here as >11321 for TOPICAL and >22222 for SOCCAR) are also those with a higher frequency of adverse events. In the TOPICAL trial, 24% of patients in ‘Poor’ health states (i.e. worse than 11321) experienced more than 2 grade 3-4 adverse events compared with 15% for patients in ‘Good’ health states (health states 11321 or better); for SOCCAR this was 66% vs. 44%.

One reason why mapping algorithms over-predict EQ-5D-3L at ‘Poor’ health states might be because treatment related toxicity is not directly captured into the mapping algorithm, resulting in estimating a QoL higher than it actually is. A similar pattern was also seen for other cut-offs which define ‘Poor’ and ‘Good’. For example, when the cut-off for ‘good’ and ‘poor’ was defined as health states 21321 (EQ-5D-3L of 0.364) and >22111 respectively, fewer patients in ‘good’ health states had AEs compared with patients with ‘poor’ health states: 17% vs. 26% of patients in ‘good’ vs. ‘poor’ health states had at least 2 adverse events in the TOPICAL trial. A similar pattern was observed for SOCCAR data. This suggests that there may be a more complex underlying mapping algorithm between EQ-5D-3L, QLQ-C30 and toxicity which might better explain the variability and prediction of EQ-5D-3L, particularly in patients with ‘Poor’ health states.

### Impact on QALY estimates

Table 4 compares observed and expected QALYs from each of the models for SOCCAR and TOPICAL separately. The Observed QALY difference was 0.051 for TOPICAL (Erlotinib vs. Placebo) and 0.164 for SOCCAR (Concurrent vs. Sequential). Predictions from the BB model generated closest QALY estimates in both trials with a mean QALY difference of 0.053 for TOPICAL and 0.162 for SOCCAR (Table 4). QALY predictions from other models ranged from 0.041 (Linear) to 0.072 (Quadratic) for TOPICAL and 0.153 (Linear) to 0.208 (Quantile).

**Table 4:** **Comparison of estimated (mean) QALY’s for all algorithms**

	**TOPICAL**	**SOCCAR**				
	**Tarceva**	**Placebo**	**Difference**	**Concurrent**	**Sequential**	**Difference**
Observed	0.35	0.30	0.051	1.31	1.15	0.164
BB	0.34	0.29	0.053	1.53	1.37	0.162
TOBIT	0.37	0.31	0.064	1.59	1.42	0.174
CLAD	0.33	0.29	0.046	1.62	1.44	0.186
Quadratic	0.42	0.34	0.072	1.92	1.73	0.196
Mixed linear	0.32	0.28	0.041	1.34	1.19	0.153
Quantile	0.38	0.31	0.070	1.42	1.62	0.208

### Adjustment for demographic variables

Several additional factors were added to the model. Although R^2^ changed slightly from 0.75 to 0.78 in TOPICAL with the inclusion of ECOG (P < 0.001) and Gender (P < 0.001), the underlying pattern of prediction shown in Table 2, Table 3 and Figure 4 did not change. Adding demographic variables does improve model fit slightly but does not have a major impact on predicted means and their standard errors (available on request from authors).

## Discussion

Superior predictive properties have been demonstrated with a non-linear BB mapping algorithm developed and tested using data from two independent lung cancer patient populations. Two mapping algorithms for different types of lung cancer patients (poor and better prognosis) have been shown to perform better than commonly used models. Either algorithm could be used, but our preference is for the one derived from the larger TOPICAL trial due to smaller uncertainty (Table 3) and better model fit (Table 2). Simulations assessed the uncertainty of mean estimates of EQ-5D-3L utilities. The degree of over-prediction of mean utilities at poorer health states was less with the BB model compared to other models. QALY estimates from models were also closer to the observed values with the BB model. Our findings confirm previous untested assertions that the relationship between the EQ-5D-3L and QLQ-C30 may be better understood with a non-linear model structure [5,9,15].

Previous mapping models have mostly used OLS forms, considered inadequate or over simplistic [6]. Most reported mapping models suffer from over-prediction at poorer health states. Previously reported models have reported R^2^ values (using QLQC-30) ranging from 0.23 to 0.83 [6]; In our study, we report similar values for these models. Rarely do models yield values of R^2^ above 70%. Other models (e.g. multinomial, ordinal) have also been used, but shown to be inadequate [5]. Estimates based on absolute deviation (CLAD, Quantile and adjusted censored models) predict patient level medians, whereas the statistic of interest is the mean. The Quadratic model takes into account non linearity (by having squared terms in the model) but is essentially a linear model (linear in parameters because the coefficients are interpreted in the same way as linear models).

Improving model fit by “discarding” or “weighting” outliers with extreme values [5] is not an optimal solution if the extreme outliers with can be modelled (rather than excluding observations). Moreover, the choice as to which variables to square and then combine with non-squared variables in quadratic models has many possibilities. For example, squaring all 15 domain scores of the QLQ-C30 is one possible choice, as is squaring 14 and have only one non-squared term remaining. Without doing a very large number of tests and increasing the type I error, it is difficult to understand the relative merits of one set of variables over another. Therefore, this can lead to some arbitrary selection of combinations of terms to improve model fit.

There are several advantages of the BB approach. The BB model applied to this data confirms some superior statistical properties in terms of accuracy and efficiency [38,41]. The clinical interpretation of the model is still reasonably clear. Clinical relevance is still important since if as some argue [45] that a generic measure is sufficient to provide both estimates of utilities and a clinical effect size of HRQoL, estimates based on odds ratios are likely to be more easily interpretable: for example the clinical relevance of a mean treatment difference of 0.012 on the EQ-5D-3L is difficult to judge. However, if this was equivalent to an odds ratio of 1.2 (a 20% improvement in HRQoL), a way of relating both clinical effects and utilities becomes possible. This can also be extended to the response domains; treatment effects can be interpreted using a ratio scale and mean differences also derived. This makes the BB a powerful and flexible mapping algorithm relevant to health economic evaluation, policy and clinical decision making.

The strength of our research lies in our approach to validating the model using independent data and extensive multivariate simulation from correlated EQ-5D-3L and QLQ-C30 data. We also explore some plausible reasons why over-prediction at the poorer health states occur by using adverse event data (collected in all trials) often ignored in mapping algorithms. It is possible that joint relationships between adverse events and EQ-5D-3L might offer a plausible explanation for over-prediction, because higher toxicity was observed in the poorer health states. Some researchers suggest that EQ-5D-3L responses have a bimodal distribution and therefore 2 separate mapping algorithms might be needed Veerstegh [25] for patients in ‘Poor’ and ‘Good’ health states. The nature of the bimodality could be explored using baseline clinical data (e.g. using baseline ECOG).

Our research has several limitations. We have not exploited the impact on results if the true values of α and β are different to the sample estimates. In our application, we set α and β so that the mean EQ-5D-3L could be modelled; other possibilities might include searching for α and β which might optimize R^2^, minimise MSE and improve predictions. Secondly, we assumed a scale of 0 to 1, which might be suitable for some disease but may not be suitable for others where states worse than death are likely to be more important. Surprisingly, even in this NSCLC population, the proportion of such cases were low. Thirdly, model validation has also been limited to lung cancer data and further testing would be useful in both lung cancer (to see whether algorithms are tumour specific) and non-lung cancer data sets (to check for generalizability). Finally, we did not compare the BB with other models such as Bayesian network models which report superior predictive properties compared to the more common models. However with such Bayesian models, the choice of the initial (prior) estimates of probability of EQ-5D-3L responses can influence the predicted utility.

There are several concerns when using mapping functions, a point repeated in previous research [5,6,15,19]. One key concern is that it is unknown whether the predicted utilities are close to the observed values unless we know both. Secondly, there are questions as to what exactly is being measured [13] or estimated because some key information in one instrument is not included in the other, particularly when predicting EQ-5D-3L from clinical measures alone. One approach might be to look at the psychometric properties of the two instruments and also check correlations. Weak correlation (Spearman’s or linear) might explain a poor mapping algorithm.

The first concern regarding mapping can be partially answered with the use of simulation by quantifying uncertainty in how well the predicted approximates the observed can be quantified as in Table 3. This does not tell us what the predicted EQ-5D-3L is actually measuring, but it is assumed that the closer the predicted values are to the observed numerically, the preferences become ‘essentially similar’. If in 90% of simulations, the observed and predicted values are close, it may be reasonable to assume that the mapping algorithm provides estimates that are measuring aspects of “essentially similar” preferences, which for practical purposes might be acceptable. Moreover, the statistical significance of several predictors might also indicate the nature of preferences; for example pain was a highly significant predictor in the model (p < 0.0001) for the BB model, hence the ‘nature’ of predicted preferences includes pain (also measured with EQ-5D-3L). If a model predicts every EQ-5D-3L perfectly, then we may wish to conclude that the model has correctly predicted the ‘essential nature’ of the preferences (ultimately contained in a single index), or remain sceptical and seek additional evidence to confirm that the ‘essential nature’ of preferences are captured by the model.

Other concerns with mapping involve time points used when developing and applying an algorithm. For example, including baseline data in a model which aims to predict post-baseline treatment differences may lead to misleading estimates. Assumptions that the rates of change in EQ-5D-3L (the coefficients of the QLQ-C30) are constant from one cancer type to another are also unlikely to hold. If baseline or demographic variables are used, the relevance of these for the target population using the algorithm will also be important. Finally, the mapping algorithm should offer reasonable clinical interpretation. In lung cancer, for example we might expect dyspnoea to be an important predictor of HRQoL. In some models, dyspnoea (Additional file 1: Table S2) was not statistically relevant for predicting EQ-5D-3L, although it is an important symptom in lung cancer patients. For increasing symptoms scores we might expect EQ-5D-3L utilities to decrease which is not always the case.

## Conclusions

The Beta-Binomial regression approach shows superior performance compared with published models in terms of predicting the observed EQ-5D-3L from QLQ-C30 in these lung cancer trials. This non-linear approach may offer advantages over existing models for mapping and as a general modelling procedure for utilities. We also confirm recent results that mapping algorithms have shown to over-estimate the HRQoL at the poorer heath states. The reasons why current algorithms persistently over-predict at poorer health states requires further interrogation, perhaps incorporating adverse event information into the models. Guidelines on using algorithms may also be useful. Mapping may be useful however there are still concerns as to whether the predicted utilities are essentially the same as the observed values.

## Consent

Written informed consent was obtained from patients for the publication of this report and accompanying images.

## Additional files

Additional file 1: Table S1. Summary Statistics of EQ-5D and QLQC30. **Table S2**: Summary of Models: Coefficients. (P-values). Additional file 2: Figure S1. Models compared in terms of patient level predictions. Additional file 3: Figure S2. Normal Probability plots (TOPICAL).

## References

[1] Guide to the methods of technology appraisals. [website]: NICE; 2004 [cited 2012 1st October]; Available from: http://www.nice.org.uk/aboutnice/howwework/devnicetech/technologyappraisalprocessguides/GuideToMethodsTA201112.jsp.

[2] Dolan P (1997). Modeling valuations for EuroQol health states. Med Care.

[3] Drummond MA (2001). Economic Evaluation in Health Care.

[4] Dunlop W, Uhl R, Khan I, Taylor A, Barton G (2012). Quality of life benefits and cost impact of prolonged release oxycodone/naloxone versus prolonged release oxycodone in patients with moderate-to-severe non-malignant pain and opioid-induced constipation: a UK cost-utility analysis. J Med Econ.

[5] Crott R, Versteegh M, Uyl-de-Groot C (2013). An assessment of the external validity of mapping QLQ-C30 to EQ-5D preferences. Qual Life Res.

[6] Brazier JE, Yang Y, Tsuchiya A, Rowen DL (2010). A review of studies mapping (or cross walking) non-preference based measures of health to generic preference-based measures. Eur J Health Econ.

[7] Barton GR, Sach TH, Jenkinson C, Avery AJ, Doherty M, Muir KR (2008). Do estimates of cost-utility based on the EQ-5D differ from those based on the mapping of utility scores?. Health Qual Life Out.

[8] Jäkel A, Plested M, Dharamshi K, Modha R, Bridge S, Johns A (2013). A systematic review of economic evaluations in second and later lines of therapy for the treatment of non-small cell lung cancer. Appl Health Econ Health Policy.

[9] Longworth L, Rowen D (2013). Value Health.

[10] Scuffham PA, Whitty JA, Mitchell A, Viney R (2008). The use of QALY weights for QALY calculations: a review of industry submissions requesting listing on the Australian Pharmaceutical Benefits Scheme 2002-4. Pharmacoeconomics.

[11] CHMP (2005). Guideline on Clinical Investigation of Medicinal Products for the Treatment of Multiple Sclerosis.

[12] Rowen D, Brazier J, Young T, Gaugris S, Craig BM, King MT, Velikova G (2011). Deriving a preference-based measure for cancer using the EORTC QLQ-C30. Value Health.

[13] Round J: *Capturing Information Loss in Estimates of Uncertainty That Arise from Mapping Algorithms.* ᅟ: Health Economists’ Study Group; 2008.

[14] Chuang LH, Whitehead SJ (2012). Mapping for economic evaluation. Br Med Bull.

[15] Crott R, Briggs A (2010). Mapping the QLQ-C30 quality of life cancer questionnaire to EQ-5D patient preferences. Eur J Health Econ.

[16] Jang RW, Isogai PK, Mittmann N, Bradbury PA, Shepherd FA, Feld R, Leighl NB (2009). Derivation of utility values from EORTC QLQ-C30 values in lung cancer. J Thorac Oncol.

[17] Kim EJ, Ko SK, Kang HY (2012). Mapping the cancer-specific EORTC QLQ-C30 and EORTC QLQ-BR23 to the generic EQ-5D in metastatic breast cancer patients. Qual Life Res.

[18] Kontodimopoulos N, Aletras VH, Paliouras D, Niakas D (2009). Mapping the cancer-specific EORTC QLQ-C30 to the preference-based EQ-5D, SF-6D, and 15D instruments. Value Health.

[19] McKenzie L, van der Pol M (2009). Mapping the EORTC QLQ C-30 onto the EQ-5D instrument: the potential to estimate QALYs without generic preference data. Value Health.

[20] Kharroubi SA, Brazier JE, Roberts J, O’Hagan A (2007). Modelling SF-6D health state preference data using a nonparametric Bayesian method. J Health Econ.

[21] Gray AM, Rivero-Arias O, Clarke PM (2006). Estimating the association between SF-12 responses and EQ-5D utility values by response mapping. Med Decis Making.

[22] Hernández Alava M, Wailoo AJ, Ara R (2012). Tails from the peak district: adjusted limited dependent variable mixture models of EQ-5D questionnaire health state utility values. Value Health.

[23] Basu A, Manca A (2012). Regression estimators for generic health-related quality of life and quality-adjusted life years. Med Decis Making.

[24] Le QA, Doctor JN (2011). Probabilistic mapping of descriptive health status responses onto health state utilities using Bayesian networks: an empirical analysis converting SF-12 into EQ-5D utility index in a national US sample. Med Care.

[25] Versteegh MM, Rowen D, Brazier JE, Stolk EA (2010). Mapping onto EQ-5D for patients in poor health. Health Qual Life Out.

[26] Paolino P (2001). Maximum likelihood Estimation of models with beta-distributed dependent variables. Polit Anal.

[27] EORTC Quality of life. EORTC:[cited 2012 10th October]; Available from: http://groups.eortc.be/qol/eortc-qlq-c30.

[28] Lee SM, Khan I, Upadhyay S, Lewanski C, Falk S, Skailes G, Marshall E, Woll PJ, Hatton M, Lal R, Jones R, Toy E, Chao D, Middleton G, Bulley S, Ngai Y, Rudd R, Hackshaw A, Boshoff C (2012). First-line erlotinib in patients with advanced non-small-cell lung cancer unsuitable for chemotherapy (TOPICAL): a double-blind, placebo-controlled, phase 3 trial. Lancet Oncol.

[29] Maguire J, Khan I, McMenemin R, O'Rourke N, McNee S, Kelly V, Peedell C, Snee M. **SOCCAR: A randomised phase II trial comparing sequential versus concurrent chemotherapy and radical hypofractionated radiotherapy in patients with inoperable stage III Non-Small Cell Lung Cancer and good performance status.** *Eur J Cancer**. 2014 Oct 7.* doi:10.1016/j.ejca.2014.07.009 10.1016/j.ejca.2014.07.009 25304298

[30] Honore B, Khan S, Powell JL (2002). Quantile regression under random censoring. J Econometrics.

[31] Koenker R (2005). Quantile Regression (Econometric Society Monographs).

[32] Khan S, Powell JL (2001). Two-step estimation of semiparametric censored regression models. J Econometrics.

[33] Powell JL (1984). Least absolute deviations estimation for the censored regression-model. J Econometrics.

[34] Kaambwa B, Billingham L, Bryan S (2013). Mapping utility scores from the Barthel index. Eur J Health Econ.

[35] Willan AR (2006). Statistical analysis of cost-effectiveness data from randomized clinical trials. Expert Rev Pharmacoecon Outcomes Res.

[36] Willan AR (2011). Sample size determination for cost-effectiveness trials. Pharmacoeconomics.

[37] Briggs AH, Claxton K, Sculpher MJ (2006). Decision Modelling for Health Economic Evaluation.

[38] Ospina R, Ferrari SLP, Cribari-Neto F (2004). A general class of zero-or-one inflated beta regression models. Comp Stat Data Analysis.

[39] Kieschnick R, McCullough BD (2003). Regression analysis of variates observed on (0,1): percentages, proportions and fractions. Stat Model.

[40] Swearingen CJ, Castro MSM, Bursac Z (2012). Inflated Beta Regression: Zero, one and Everything in Between.

[41] Ospina R, Ferrari SLP (2012). A general class of zero-or-one inflated beta regression models. Comput Stat Data Anal.

[42] Ferrari SLP, Cribari-Neto F (2004). Beta regression for modelling rates and proportions. J Appl Stat.

[43] Fleishman AI (1978). A method of simulating non-normal distributions. Psychometrika.

[44] Pourahmadi M, Daniels MJ, Park T (2007). Simultaneous modelling of the Cholesky decomposition of several covariance matrices. J Multivariate Anal.

[45] Kind P: **Measuring the value of quality of life in cancer: an index based on EORTC QLQ-C30 presentation (abstract), ASCO 2005 Annual Meeting.** *J Clin Oncol* 2005, **29**(16S, Part I and II).

