# Supplementary material for: A non-linear beta-binomial regression model for mapping EORTC QLQ- C30 to the EQ-5D-3L in lung cancer patients: a comparison with existing approaches
Source: Health Qual Life Outcomes. 2014 Nov 12;12:163. doi: 10.1186/s12955-014-0163-7 (PMC4234877; doi:10.1186/s12955-014-0163-7)
Supplement: Additional file 1: Table S1. — Summary Statistics of EQ-5D and QLQC30. Table S2: Summary of Models: Coefficients. (P-values). [file 12955_2014_163_MOESM1_ESM.doc]

**Additional file 1**

**Table S1: Summary Statistics of EQ-5D and QLQC30**

|  | ***TOPICAL (N=670)*** | | | | | ***SOCCAR (N=130)*** | | | | |
| --- | --- | --- | --- | --- | --- | --- | --- | --- | --- | --- |
|  | **Baseline** | **3 months** | **6 months** | **12 months** | **Overall** | **Baseline** | **3 months** | **6 months** | **12 months** | **Overall** |
|  | Mean (SD) | Mean (SD) | Mean (SD) | Mean (SD) | Mean (SD) | Mean (SD) | Mean (SD) | Mean (SD) | Mean (SD) | Mean (SD) |
| **EQ-5D** | 0.56 (0.30) | 0.61 (0.30) | 0.66 (0.26) | 0.58 (0.36) | 0.61 (0.29) | 0.79 (0.17) | 0.71 (0.24) | 0.71 (0.25) | 0.71 (0.33) | 0.75 (0.23) |
| **PF** | 51.53( 26.44) | 51.66 (24.91) | 54.00 (25.41) | 54.29 (29.14) | 54.15 (26.30) | 86.19 (14.50) | 72.06 (23.78) | 79.11 (43.57) | 98.52 (84.21) | 78.60 (36.53) |
| **RF** | 45.18 (36.58) | 45.70 (34.26) | 51.36 (32.79) | 51.41 (33.94) | 49.18 (34.67) | 80.84 (24.40) | 63.11 (33.28) | 72.67 (49.22) | 90.37 (89.43) | 71.28 (43.04) |
| **EF** | 70.96 (24.13) | 73.17 (24.02) | 75.69 (24.49) | 74.58 (27.18) | 73.89 (24.41) | 73.41 (23.68) | 71.91 (28.30) | 80.33 (46.39) | 91.30 (91.60) | 77.06 (42.23) |
| **FI** | 75.47 (25.75) | 77.24 (25.10) | 78.15 (24.27) | 68.36 (27.28) | 77.17 (24.41) | 85.85 (26.84) | 78.28 (24.15) | 88.22 (42.34) | 103.33 (83.08) | 83.13 (42.32) |
| **SF** | 59.47 (36.00) | 64.71 (33.76) | 68.69 (31.76) | 64.94 (31.79) | 66.90 (32.40) | 84.52 (25.58) | 64.79 (36.79) | 76.67 (47.77) | 92.59 (91.05) | 74.55 (45.17) |
| **QL** | 47.60 (25.27) | 51.64 (23.03) | 55.29 (22.84) | 52.68 (23.49) | 52.27 (23.25) | 71.36 (18.48) | 56.93 (22.93) | 59.78 (33.09) | 55.56 (59.96) | 63.75 (29.65) |
| **FA** | 53.72 (28.59) | 51.45 (29.59) | 47.41 (28.67) | 45.20 (28.03) | 48.52 (28.89) | 26.42 (25.47) | 46.44 (30.36) | 35.41 (50.09) | 13.33 (89.88) | 33.94 (42.20) |
| **NV** | 14.14 (22.61) | 11.89 (19.55) | 8.56 (15.24) | 9.89 (16.41) | 10.65 (18.81) | 6.82 (25.92) | 17.42 (26.70) | 11.78 (43.22) | 12.96 (86.84) | 10.58 (37.26) |
| **PA** | 32.22 (32.97) | 26.11 (29.97) | 24.89 (28.54) | 26.84 (32.02) | 26.00 (29.70) | 17.45 (22.41) | 23.41 (26.32) | 23.33 (49.32) | 5.93 (91.09) | 20.66 (41.47) |
| **DY** | 55.72 (33.49) | 50.40 (32.56) | 49.89 (34.32) | 52.54 (31.07) | 49.76 (32.92) | 29.66 (33.92) | 30.71 (30.66) | 30.67 (49.85) | 14.81 (90.61) | 31.57 (45.98) |
| **SL** | 33.71 (33.83) | 32.28 (34.47) | 24.49 (29.54) | 31.61 (33.87) | 29.03 (32.67) | 27.03 (31.63) | 32.96 (33.52) | 22.22 (48.81) | 8.15 (89.66) | 24.00 (42.70) |
| **AP** | 44.07 (38.14) | 42.15 (36.44) | 32.66 (34.22) | 32.20 (32.73) | 36.78 (35.57) | 16.54 (25.50) | 30.34 (47.31) | 24.44 (49.12) | 3.70 (89.39) | 20.47 (43.61) |
| **CO** | 28.12 (34.81) | 21.95 (29.00) | 15.86 (24.87) | 20.83 (26.64) | 20.33 (28.21) | 13.76 (22.42) | 37.45 (47.63) | 11.56 (45.02) | 10.00 (92.93) | 18.64 (46.78) |
| **DI** | 19.59 (30.71) | 20.57 (30.91) | 16.32 (25.49) | 16.67 (25.93) | 17.14 (27.54) | 3.70 (10.52) | 7.12 (19.77) | 2.22 (38.88) | 20.00 (99.75) | 6.06 (40.62) |
| **CF** | 14.36 (25.35) | 9.57 (19.95) | 7.43 (18.98) | 12.07 (24.74) | 10.00 (21.02) | 20.11 (31.86) | 23.22 (41.26) | 19.56 (50.85) | 10.74 (86.89 | 20.44 (49.53) |

Physical Function (PF), Role Function (RF), Emotional Function (EF), Cognitive Function (CF), Social Functioning (SF); Fatigue (FA), Nausea & Vomiting (NV), Pain (PA), Dyspnoea (DY), Insomnia (IN), Appetite Loss (AL), Constipation (CO), Diarrhoea (DI), Financial Problems (FI); Global Health Status Score (QL).

**Table S**2: Summary of Models: Coefficients (P-values)

| ***QLQ-C30*** | ***Linear Mixed*** | ***TOBIT*** | ***Quadratic#*** | ***Quantile*** | ***CLAD*** | ***Beta*** |
| --- | --- | --- | --- | --- | --- | --- |
| **PF** | 0.0029, 0.0019  (0.0001, <0.0001) | 0.0034, 0.0022  (<0.0001, <0.0001) | 0.0061, 0.00016  (<0.0001,0.8247) | 0.0028, 0.0022  (<0.0001, <0.0001 | 0.0028 , 0.0016  (<0.0001, <0.0001 | 0.518, 0.260  (<0.0001, <0.0001) |
| **RF**  Physical  Function | 0.0010,0.0010  (<0.0001, <0.0001) | 0.0009,0.0012  (<0.0001, <0.0001) |  | 0.0006,0.0007  (0.0150, 0.0176) | 0.0008, 0.0008  (<0.0001, 0.0012) | 0.108,0.2340  (0.0075, <0.0001) |
| **EF** | 0.0015, 0.0021  (0.0008, <0.0001) | 0.0019, 0.0025  (<0.0001, <0.0001) | 0.00409, 0.00042  (<0.0001, 0.5028) | 0.0019, 0.0026  (<0.0001, <0.0001 | 0.0017, 0.0028  (<0.0001, <0.0001) | 0.067, 0.379  (<0.0001, <0.0001) |
| **SF** | 0.0009,0.00010  (<0.0001, <0.0001) | 0.0009,0.0011  (<0.0001, <0.0001) | 0.00172,0.00152  (0.0013, <0.0001) | 0.0016,0.0005  (<0.0001, 0.0039) | 0.0015, 0.0007  (0.0015, 0.0086) | 0.029, 0.257  (0.0452, <0.0001) |
| **CF** | -0.0003,-0.0002  (<0.0001, 0.9293) | -0.0003, -0.0007  (0.4422, 0.8041) |  | 0.0003,0.0001  (0.2143, 0.6266) | 0.0003, -0.0004  (0.0468, 0.8884) | 0.067, 0.061  (<0.0001, <0.0001) |
| **FA** | 0.0004,0.0001  (0.0163, 0.8389) | 0.0005, -0.00021  (0.0513, 0.5763) |  | 0.0008,-0.0004  (0.0244, 0.1797) | 0.0007, -0.0005  (0.0114, 0.3422) | 0.064, -0.012  (0.0122, 0.8299 |
| **NV** | -0.0002,0.0004  (0.3131, 0.1281) | -0.0001,0.0005  (0.5699, 0.0715) |  | -0.0001,0.0003  (0.6821, 0.3951) | 0.00001, 0.0004  (0.1922, 0.0998) | 0.011, 0.062  (0.6613, 0.0875) |
| **PA** | 0.0019, 0.0025  (<0.0001, <0.0001) | -0.0029, -0.0017  (<0.0001, <0.0001 | -0.0030,-0.0018  (<0.0001, <0.0001 | -0.0029, -0.0020  (<0.0001, <0.0001) | -0.0024, -0.0018  (<0.0001, 0.0019) | -0.496, -0.235  (<0.0001, <0.0001) |
| **DY**  Symptoms | 0.0004,0.00001  (00.330, 0.9469) | 0.0004,-0.0002  (0.0361, 0.3748) |  | 0.0003,-0.0001  (0.0670, 0.4068) | 0.0002, 0.0001  (0.0466, 0.5325) | 0.065, 0.0088  (0.0219, 0.8316) |
| **SL** | -0.0004,-0.0004  (0.0161, 0.0044) | -0.0004,-0.0006  (0.0053, 0.0059) | -0.00085, -0.00087  (0.0249, 0.0017) | -0.0004,-0.0005  (0.0410, 0.0032) | -0.0003, -0.0004  (0.0333, 0.0024) | -0.062, -0.102  (0.0195, 0.0036) |
| **AP** | 0.0001,-0.0002  (0.7427, 0.1192) | 0.00007,-0.0002  (0.6337, 0.3325) |  | 0.0001,-0.0001  (0.7616, 0.6294) | 0.00001, -0.00007  (0.6257, 0.5998) | 0.0265, 0.0017  (0.3421, 0.9660) |
| **CO** | -0.0003,-0.0001  (0.0602, 0.0882) | -0.0004,0.0002  (0.0101, 0.2632) | -0.00036, 0.0062  (0.0177, 0.2514) | -0.0002,0.0003  (0.3031, 0.1062) | -0.0002, 0.0004  (0.2145, 0.1145) | -0.082, 0.026  (0.0010, 0.4835) |
| **DI** | -0.0002,0.0007  (0.9213, 0.0060) | -0.0001,0.0009  (0.5608, 0.0009) | -0.00062,-0.00029  (0.1257, 0.4490) | -0.0001,0.0001  (0.8258, 0.8379) | -0.0004, 0.0009  (0.7589, 0.4417) | -0.017,0.051  (0.4614, 0.1574) |
| **FI**  Finance and Overall QoL | 0.0004,-0.0001  (0.0076, 0.0002) | -0.0007,0.00037  (0.0222, 0.0418) |  | 0.0004,0.0001  (0.0325, 0.4257) | -0.0039, 0.0008  (0.0187, 0.0587) | -0.005, 0.062  (0.0039, 0.0715) |
| **QL** | 0.0014, 0.0013  (0.0163, 0.0002) | 0.0018, 0.00018  (<0.0001, <0.0001 |  | 0.0014, 0.0009  (<0.0001, <0.0001 | 0.0013, 0.0014  (<0.0001, <0.0001 | 0.237, 0.224  (<0.0001, <0.0001) |
|  |  |  |  |  |  |  |

#For Quadratic, P-values for PF^2^, PF^2^, PF^2^, PF^2^, PF^2^ were (0.0002, <0.0001); (0.003, 0.002); (0.503, 0.261); (0.286, 0.034); (0.251, 0.015) respectively
