# Supplementary material for: A non-linear beta-binomial regression model for mapping EORTC QLQ- C30 to the EQ-5D-3L in lung cancer patients: a comparison with existing approaches
Source: Health Qual Life Outcomes. 2014 Nov 12;12:163. doi: 10.1186/s12955-014-0163-7 (PMC4234877; doi:10.1186/s12955-014-0163-7)

**Additional file 2: Figure S1: Models compared in terms of patient level predictions**

1. **TOPICAL**
2. **SOCCAR**


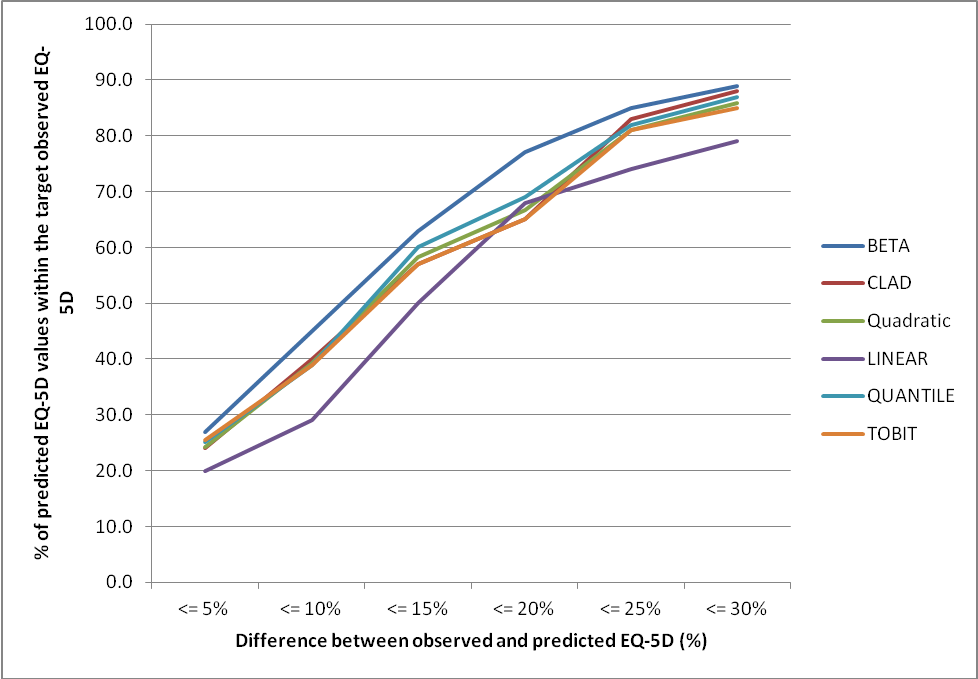

Supplement: Additional file 2: Figure S1. — Models compared in terms of patient level predictions. [file 12955_2014_163_MOESM2_ESM.docx]
