# Supplementary material for: A non-linear beta-binomial regression model for mapping EORTC QLQ- C30 to the EQ-5D-3L in lung cancer patients: a comparison with existing approaches
Source: Health Qual Life Outcomes. 2014 Nov 12;12:163. doi: 10.1186/s12955-014-0163-7 (PMC4234877; doi:10.1186/s12955-014-0163-7)
Supplement: Additional file 3: Figure S2. — Normal Probability plots (TOPICAL). [file 12955_2014_163_MOESM3_ESM.doc]

Additional file 3: **Figure S2: Normal Probability plots (TOPICAL)**


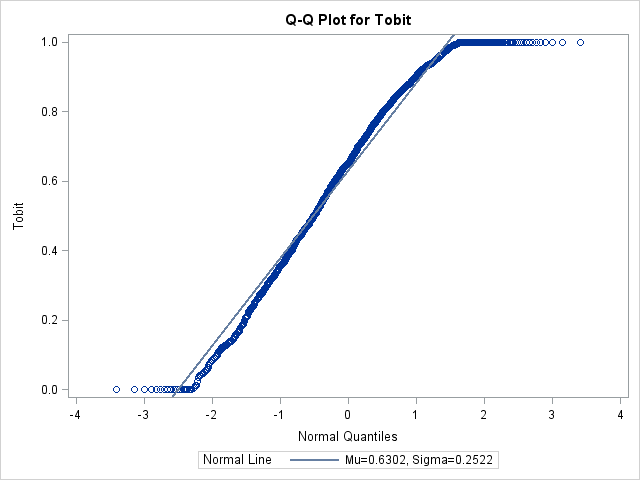


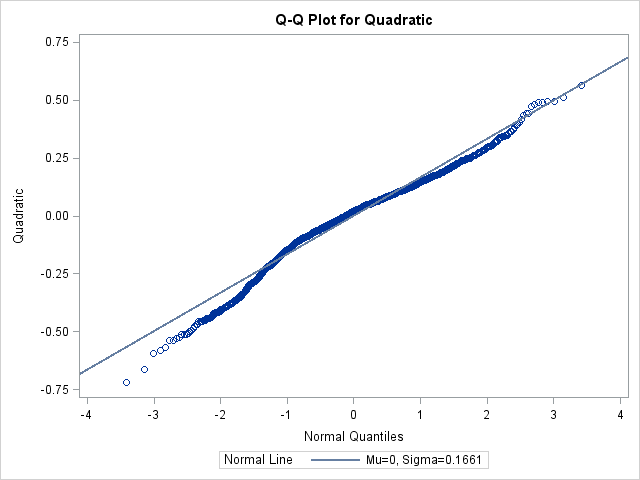

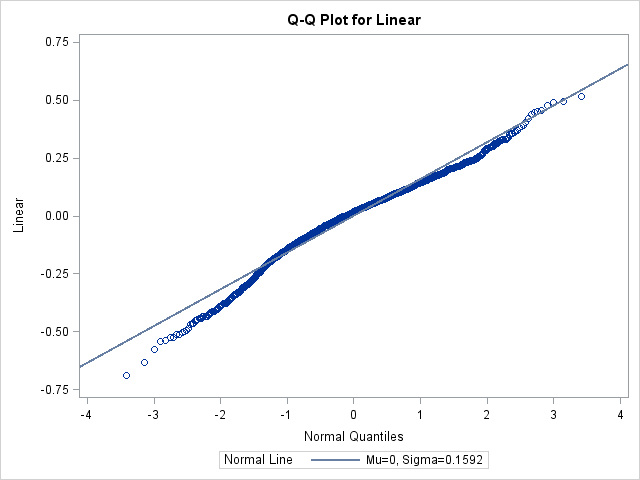


TOBIT

Quadratic

Linear


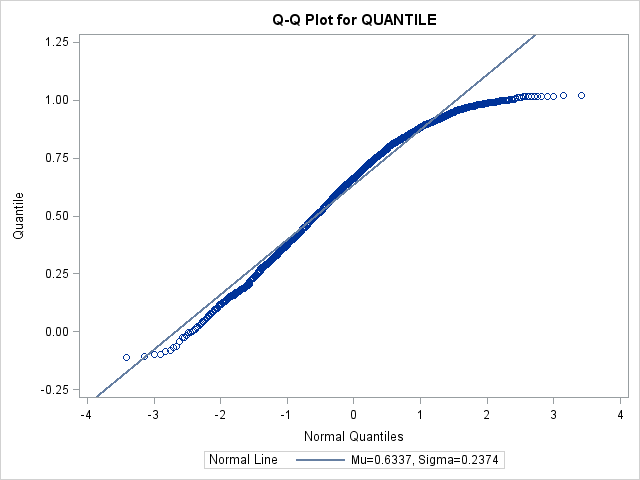


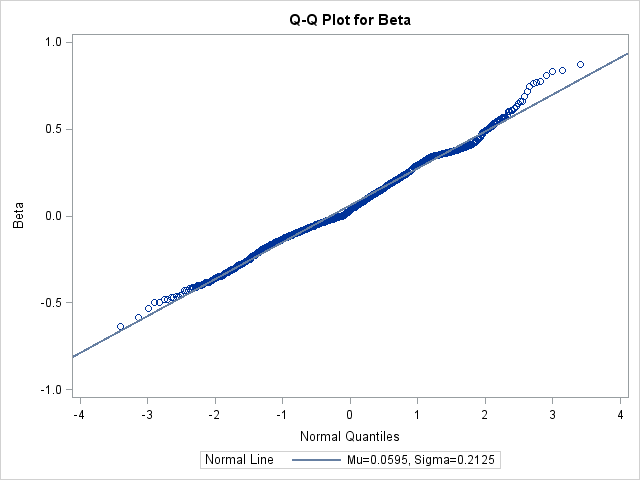

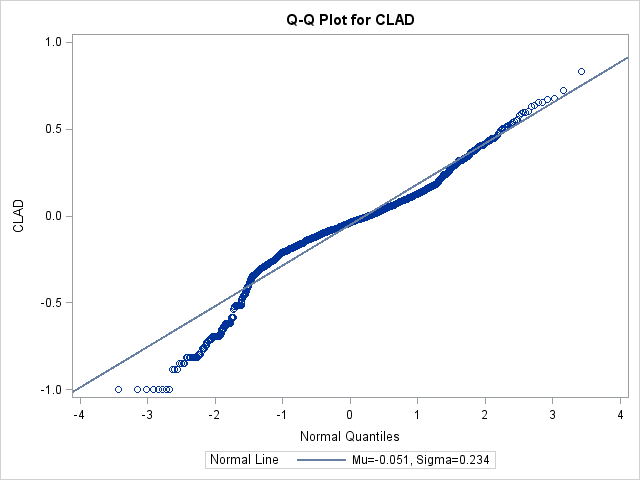


Beta Binomial

Quantile

CLAD
